# Bleeding after endobronchial biopsy: sometimes frightening, often safe, always careful

**DOI:** 10.1186/s12890-023-02331-9

**Published:** 2023-01-25

**Authors:** Saibin Wang, Qian Ye

**Affiliations:** 1 Department of Pulmonary and Critical Care Medicine, Jinhua Municipal Central Hospital, No. 365, East Renmin Road, Jinhua, 321000 Zhejiang Province China; 2 Department of Medical Records Quality Management, Jinhua Municipal Central Hospital, No. 365, East Renmin Road, Jinhua, 321000 Zhejiang Province China

**Keywords:** Bronchoscopy, Biopsy, Lung cancer, Bleeding

## Abstract

We explain to Dr. Govindasaami’s several comments on our published article “Association between blood pressure and the risk of biopsy-induced endobronchial hemorrhage during bronchoscopy”.

Author response

Dr. Wahidi et al. collected a survey from 158 pulmonologists attending the American College of Chest Physicians’ annual meeting in 2001, and concluded that the effect of pulmonary hypertension on transbronchial lung biopsy (TBLB) is still not clearly defined [1]. Hypothetically, the increased perfusion pressure in the pulmonary capillary bed may increase the risk of bleeding following TBLB [1]. However, it should be noted that endobronchial biopsy (EBB), which is the biopsy method used in our study [2], is different from TBLB. TBLB is usually used to collect samples of peripheral lung lesions, diffuse lesions, or infiltrative lesions around the lung, meaning that the lesions commonly receive blood supply from both bronchial and pulmonary artery. In addition, TBLB is generally performed with or without X-ray guidance, and it is usually difficult to see the lesions under the direct view of the bronchoscope. Nevertheless, in our study, lung cancer samples were collected using EBB. On the one hand, the main blood supplies to lung cancer come from the bronchial arteries [3, 4], thus, the pulmonary artery pressure has anatomically little influence with EBB-induced bleeding. On the other hand, EBB is commonly performed under the direct vision of the bronchoscope (Fig. 1). Therefore, EBB is more effective in avoiding the area of the lesion with greater vascularity than TBLB. However, the patients with uncontrolled pulmonary arterial hypertension, as well as congestive heart failure, were excluded in advance from our study by the anesthesiologist because all these patients were subjected to bronchoscopy under general anesthesia.

**Fig. 1:**
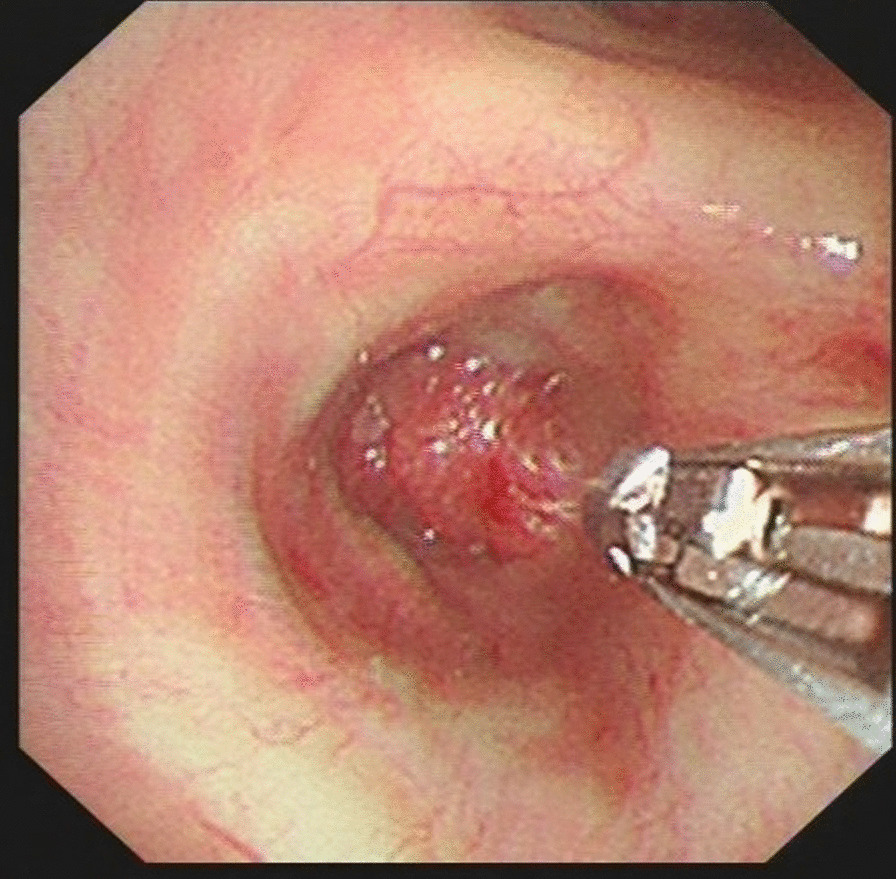
Endobronchial biopsy is performed under the direct vision of bronchoscope

In case of a previous history of hemoptysis before bronchoscopy, Faiz et al. were concerned about the incidence of bleeding complications with flexible bronchoscopy in a group of special patients with cancer combined with thrombocytopenia [5]. Indeed, these patients with severe thrombocytopenia requiring platelet transfusion support, or under anticoagulant drugs, are themselves prone to spontaneous bleeding even without bronchoscopy. Therefore, Faiz et al. excluded the procedures of endobronchial or transbronchial biopsies, brushings, and transbronchial fine needle aspirations. However, all the patients included in our study received EBB, but patients with thrombocytopenia, and those under anticoagulant drugs were not included in our study. A previous history of hemoptysis as a predictor of risk of EBB bleeding was not observed in our study.

In case the lesion is located in proximity to a vessel (like superior vena cava syndrome), the vascularity of the lesion may increase the risk of biopsy bleeding; although I agree, I think this consequence is more probable in the TBLB procedure than in the EBB.

One limitation of our study, indicated in our previous article [2], was that all the procedures were performed under general anesthesia. I agree with you that the bleeding risk might not be the same as under local anesthesia/mild sedation. Therefore, the results of our analysis might not be suitable for extrapolation to the patients undergoing EBB without general anesthesia [2].

As for the bleeding and hemostasis after EBB, although no serious bleeding eventually occurred in our study, not all the bleeding was controlled by bronchoscopy itself. Actually, approximately 13% of the patients in our study experienced refractory bleeding followed EBB. Consequently, further hemostatic measures such as argon plasma coagulation, electrocoagulation, intravenous hemostatic drug (vasopressin/hemocoagulase) were requisite after the failure of the initial intrabronchial instillation of 4 °C physiological saline or/and diluted (1:10000) adrenalin for hemostasis.

Of note, the bleeding discussed in our study is referred to post-EBB bleeding, which means that no bleeding in the airway occurred before the biopsy. We usually take 3–5 specimens (7–10 samples in recent years, mainly to carry out the molecular detection of the tumor) per lesion in our study. The worst outcome experienced during EBB was the occurrence of a significant bleeding after one biopsy; however, the bleeding did not substantially affect the histopathological diagnosis as long as a satisfactory sample was taken.

## Data Availability

The datasets used in the study are available from the corresponding author on reasonable request.

## Abbreviations

EBB: Endobronchial biopsy
TBLB: Transbronchial lung biopsy
